# The cryo-EM structure of the SF3b spliceosome complex bound to a splicing modulator reveals a pre-mRNA substrate competitive mechanism of action

**DOI:** 10.1101/gad.311043.117

**Published:** 2018-02-01

**Authors:** Lorenzo I. Finci, Xiaofeng Zhang, Xiuliang Huang, Qiang Zhou, Jennifer Tsai, Teng Teng, Anant Agrawal, Betty Chan, Sean Irwin, Craig Karr, Andrew Cook, Ping Zhu, Dominic Reynolds, Peter G. Smith, Peter Fekkes, Silvia Buonamici, Nicholas A. Larsen

**Affiliations:** 1 Beijing Advanced Innovation Center for Structural Biology, Tsinghua-Peking Joint Center for Life Sciences, School of Life Sciences, Tsinghua University, Beijing 100084, China;; 2 H3 Biomedicine, Inc., Cambridge, Massachusetts 02139, USA

**Keywords:** cancer, drug discovery, single particle cryo-EM, spliceosome

## Abstract

Somatic mutations in spliceosome proteins lead to dysregulated RNA splicing and are observed in a variety of cancers. These genetic aberrations may offer a potential intervention point for targeted therapeutics. SF3B1, part of the U2 small nuclear RNP (snRNP), is targeted by splicing modulators, including E7107, the first to enter clinical trials, and, more recently, H3B-8800. Modulating splicing represents a first-in-class opportunity in drug discovery, and elucidating the structural basis for the mode of action opens up new possibilities for structure-based drug design. Here, we present the cryogenic electron microscopy (cryo-EM) structure of the SF3b subcomplex (SF3B1, SF3B3, PHF5A, and SF3B5) bound to E7107 at 3.95 Å. This structure shows that E7107 binds in the branch point adenosine-binding pocket, forming close contacts with key residues that confer resistance upon mutation: SF3B1^R1074H^ and PHF5A^Y36C^. The structure suggests a model in which splicing modulators interfere with branch point adenosine recognition and supports a substrate competitive mechanism of action (MOA). Using several related chemical probes, we validate the pose of the compound and support their substrate competitive MOA by comparing their activity against both strong and weak pre-mRNA substrates. Finally, we present functional data and structure–activity relationship (SAR) on the PHF5A^R38C^ mutation that sensitizes cells to some chemical probes but not others. Developing small molecule splicing modulators represents a promising therapeutic approach for a variety of diseases, and this work provides a significant step in enabling structure-based drug design for these elaborate natural products. Importantly, this work also demonstrates that the utilization of cryo-EM in drug discovery is coming of age.

## Abstract (precis)

In this study, Finci et. al. present the cryo-EM structure of the SF3b subcomplex (SF3B1, SF3B3, PHF5A, and SF3B5), part of the U2 snRNP, bound to E7107 at 3.95 A. The structure suggests a model in which splicing modulators interfere with branch point adenosine recognition and supports a substrate competitive mechanism of action.

SF3B1, U2AF1, SRSF2, and ZRSR2 are components of the splicing machinery that are frequently mutated in cancer (Yoshida and Ogawa 2014). Recurring hot spot mutations are found in myelodysplastic syndromes (Yoshida et al. 2011), chronic lymphocytic leukemia (Landau et al. 2013), chronic myelomonocytic leukemia (Patnaik et al. 2013), uveal melanoma (Furney et al. 2013), skin melanoma (Kong et al. 2014), and breast (Ellis et al. 2012) and pancreatic cancers (Biankin et al. 2012). Spliceosome mutations are mutually exclusive and heterozygous and lead to missplicing of pre-mRNA to form aberrant transcripts. Some of these transcripts may be targeted for nonsense-mediated decay, leading to their down-regulation (Darman et al. 2015; Kim et al. 2015; Herdt et al. 2017), while others could be translated into aberrant proteins. Together, these may account for the “spliceosome sickness” observed in these genetic backgrounds (Darman et al. 2015) and present a potential vulnerability that could be targeted by splicing modulators in a therapeutic setting. The first splicing modulator to enter the clinic was E7107 (Hong et al. 2014), and, recently, H3B-8800 has entered phase 1 clinical trials (Seiler et al. 2018).

SF3B1 is a central component of the SF3b subcomplex in the U2 small nuclear RNP (snRNP) and plays a pivotal role in the early stages of spliceosome assembly and branch point adenosine (BPA) recognition (Fig. 1A; Gozani et al. 1998). SF3B1 recognizes the BPA in the early A complex and ultimately delivers it near the catalytic core found in the B^act^ complex (Yan et al. 2016; Plaschka et al. 2017). The departure of the SF3b subcomplex then enables the first chemical transformation, in which the BPA attacks the 5′ splice site (SS) to form the intron lariat observed in the C complex (Lardelli et al. 2010). Once liberated, the 5′ exon attacks the 3′ SS, releasing the final ligated product (Fig. 1A). E7107 is one of several natural product-like compounds known to interfere with splicing (Fig. 1B; Effenberger et al. 2016). Resistance and cross-linking studies show that these modulators interact with the SF3b complex in the BPA-binding pocket (Kotake et al. 2007; Yokoi et al. 2011; Teng et al. 2017). This binding event reduces the stability of early “A complex” formation (Fig. 1A) by weakening the interaction between the U2 snRNA and the pre-mRNA (Roybal and Jurica 2010; Corrionero et al. 2011; Folco et al. 2011; Bonnal et al. 2012; Vigevani et al. 2017). However, the precise structural basis for how these modulators interact with SF3B1 is not known.

**Figure 1.**
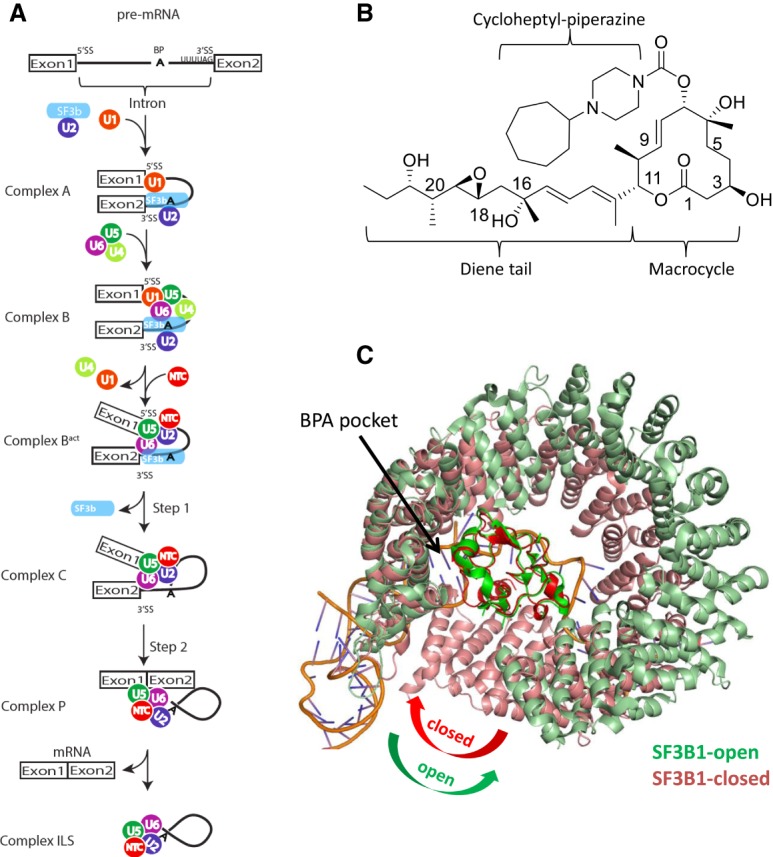
(*A*) Overview of splicing. The SF3b complex is part of the U2 snRNP and plays a central role in branch point recognition. The departure of SF3B1 is necessary for the first step of splicing, where the BPA attacks the 5′ SS. Exon ligation occurs in the second step of splicing, which is followed by product release. (*B*) The chemical structure of E7107. (*C*) The two known conformations for SF3B1. The yeast structure from the B^act^ complex bound to RNA shows a closed conformation (light red), while the apo human structure is in an open conformation (light green). The overlay was generated using the central PHF5A (dark red and dark green) as a frame of reference.

SF3B1 is intimately associated with three other proteins: PHF5A, SF3B3, and SF3B5 (Yan et al. 2016). Together, these proteins form a core scaffolding complex of ∼250 kDa. SF3B1 is composed of some 20 HEAT repeats that form a superhelical spiral of one turn that wraps around PHF5A (Fig. 1C). In this complex, the polypyrimidine tract of the pre-mRNA forms nonspecific electrostatic interactions with the N-terminal HEAT repeats (nos. 5 and 6), and the BPA is embedded in a specificity pocket around the C-terminal HEAT repeats (nos. 15–17) (Fig. 1C). These structures suggest a model in which SF3B1 acts like a molecular caliper that demarks the BPA on one side and the 3′ SS on the other and facilitates the assembly of other splicing factors for subsequent steps in the reaction. An apo crystal structure of this four-protein complex was determined to 3.1 Å, largely confirming the molecular organization revealed by the cryogenic electron microscopy (cryo-EM) structure (Cretu et al. 2016). Interestingly, comparison of the apo crystal structure with the B^act^ cryo-EM structure shows that there is significant flexibility in SF3B1 (Fig. 1C). The apo conformation is open, while the RNA-bound structure is in a more compact and closed conformation. Notably, the isolated four-protein complex binds to compounds (Teng et al. 2017), and the details of those interactions with E7107 are elucidated here at a resolution of 3.95 Å using single-particle cryo-EM.

Recent technological advancements in single-particle cryo-EM are leading to the elucidation of near-atomic structures of protein complexes and are responsible for the current “resolution revolution” (Merk et al. 2016). The prospect that cryo-EM can determine near-atomic-resolution structures is transforming the landscape of structural biology, and the potential application to drug discovery is tantalizing (Merk et al. 2016). Cryo-EM also is advantageous for determining the conformation of macromolecules in solution, unfettered by crystal contacts. Together, cryo-EM and crystallography are therefore highly complementary approaches for structural analysis. The advent of single-particle cryo-EM has provided a breakthrough in elucidating many of the various spliceosome complexes at atomic resolution (Shi 2017). Here, we explore the utilization of single-particle cryo-EM as an emerging tool for the study of protein–ligand interactions that will facilitate drug discovery at the interface of chemistry and biology, focusing on the spliceosome complex SF3b core bound to the splicing modulator E7107.

## Results

### Protein purification and compound-binding data

The cryo-EM structure of the B^act^ complex showed that the C-terminal portion of SF3B1 forms a tight complex with three other proteins: SF3B3, PHF5A, and SF3B5 (Yan et al. 2016). The four-protein complex composed of the HEAT repeats of SF3B1 (residues 454–1304) as well as full-length SF3B3, SF3B5, and PHF5A was coexpressed and purified from insect cells. This complex is necessary and sufficient for compound binding in a scintillation proximity assay (SPA) format (Teng et al. 2017). Moreover, we applied microscale thermophoresis (MST) as an orthogonal technology to interrogate direct compound binding in a homogeneous assay format and found that the complex binds E7107 with a *K*_D_ of ∼3.6 nM (Supplemental Fig. S1), in good agreement with the SPA data (Teng et al. 2017). The PHF5A^Y36C^ mutant complex was not able to bind the compound. These data are also consistent with IC_50_ values obtained from in vitro splicing assays (Teng et al. 2017) and reported GI_50_ values from various cell line models (Kotake et al. 2007; Teng et al. 2017). Therefore, these data substantiate that the subcomplex is a suitable surrogate system for further studies to interrogate the structural basis for splicing modulation.

### Structure determination

**Figure 2.**
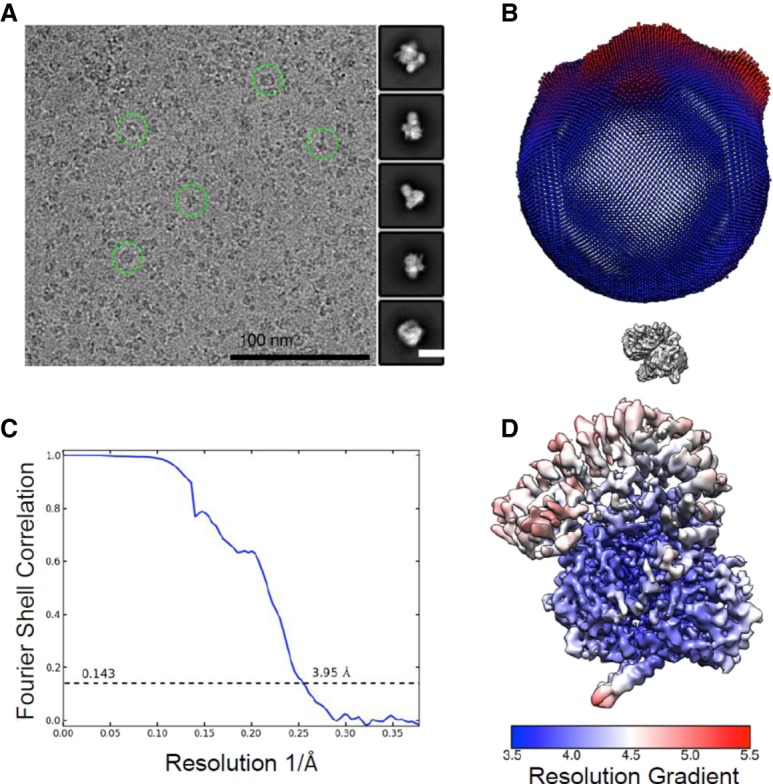
Cryo-EM analysis of the SF3b subcomplex. (*A*, *left* panel) A representative electron micrograph of SF3b with some typical particles marked by green circles. (*Right* panel) Representative 2D class averages of the particles. Bar, 100 nm. (*B*) The angular distribution of the final reconstruction. Each column represents one view, and the size of the column is proportional to the number of particles in that view. (*C*) The gold standard FSC curve for the three-dimensional (3D) reconstruction of SF3b. (*D*) Local resolution variations of the EM reconstruction. The resolution map was estimated with RELION 2.0.

Recently, cryo-EM has been gaining momentum for determining large structures bound to small molecules (Subramaniam et al. 2016). For this study, we chose E7107 because of its combination of large molecular weight and single-digit nanomolar potency. The drug-bound subcomplex was imaged on a Titan Krios (FEI) electron microscope equipped with a K2 summit camera and a LS-Quantum energy filter (Fig. 2A). A total of 900,715 particles was selected from 3371 micrographs and sorted by two-dimensional (2D) classification (Fig. 2). Reconstruction of 280,768 particles yielded an EM map of the SF3b subcomplex at an average resolution of 3.95 Å, based on the gold standard Fourier shell correlation (FSC) value of 0.143 (Fig. 2; Supplemental Fig. S2; Supplemental Table S1). One of the major challenges for this data processing campaign was a particle orientation bias, which was overcome by culling these excess particles from the final data set. The starting model for real space refinement was the apo crystal structure of SF3B1 (Protein Data Bank [PDB] ID 5IFE) (Cretu et al. 2016). After an initial round of refinement, there was a clear region of additional unexplained density located at the interface of SF3B1 and PHF5A and immediately proximal to the known resistance mutations SF3B1^K1071E^, SF3B1^R1074H^, SF3B1^V1078A/I^, and PHF5A^Y36C^ (Fig. 2B; Yokoi et al. 2011; Teng et al. 2017). The large overall size of the connected density is consistent with the size and shape of E7107. This provides compelling evidence that this is indeed the binding pocket for E7107.

### Modeling of E7107

**Figure 3.**
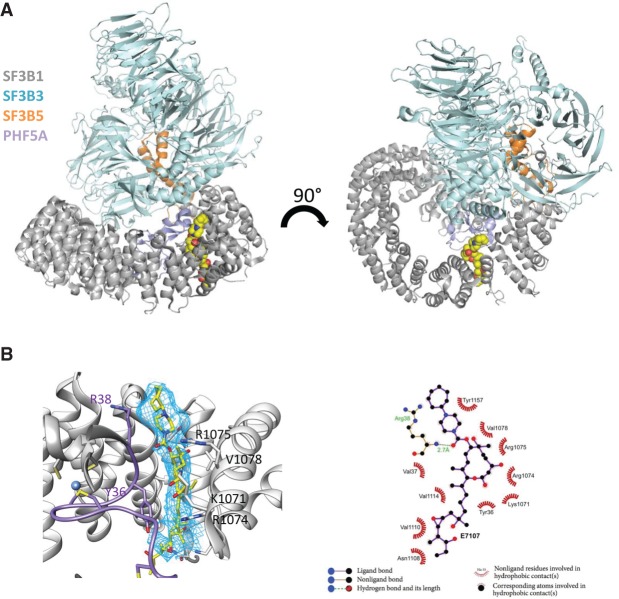
(*A*) The overall structure of the four-protein complex. HEAT repeats of SF3B1 are shown in gray, PHF5A is in light purple, SF3B3 is in cyan, and SF3B5 is in orange. E7107 is rendered as space-filling spheres colored by atom type. (*B*) The cryo-EM map shows additional density in the BPA-binding pocket. The map is contoured at 4 sec. Residue interactions are shown in a 2D schematic.

Based on this additional density, we modeled in E7107 using starting coordinates obtained from the small molecule crystal structure. The compound was docked into this density with only minor adjustments to dihedrals in the diene tail and cycloheptyl-piperazine. The conformation of the macrocycle was not altered. The cryo-EM map shows that E7107 binds at the interface of SF3B1 and PHF5A. SF3B1 encircles PHF5A and, at the interface of the HEAT repeats 15–17, forms a deep channel from the top to the bottom face, where the BPA binds (Fig. 3A). E7107 fills this channel like a plug. The cryo-EM map for the macrocycle and the cycloheptyl-piperazine are well defined, with the diene tail being slightly disordered in the terminal solvent-exposed region (Fig. 3B).

The binding pocket is located at the interface of the two HEAT repeats (HR15–HR17) from SF3B1 and PHF5A (Fig. 3B). The binding pocket is hydrophobic in nature (Fig. 3B) with the exception of residues R1074 and R1075. Residues V1110, V1114, L1066, and V1078 from SF3B1 and residue Y36 from PHF5A make interactions with the compound (Fig. 3B). The macrocycle is positioned in part by the interactions with positive-charged residues R1074, R1075, and K1071 of SF3B1 at the periphery of the channel. The cycloheptyl-piperazine is sandwiched between SF3B1^Y1157^ and PH5A^R38^ (Fig. 3B).

Compared with the previously reported apo complex crystal structure (PDB ID 5IFE) (Cretu et al. 2016), the overall conformation of the four proteins is similar after compound binding (Supplemental Fig. S3). There are only small local differences between the two structures, indicating that the modulator binding does not induce the major global conformational changes to the SF3b complex that RNA binding does. The root-mean-square deviation (RMSD) values between the different protein complexes are 1.02 Å for SF3B5 (chain B), 1.45 Å for SF3B1 (chain C), 1.11 Å for PH5a (chain D), and 1.30 Å for SF3B3 (chain A), as calculated by DynDom (Lee et al. 2003). These results show that the compound can bind in the pre-mRNA-unbound form or the so-called open conformation of the complex, which may have mechanistic implications, since this conformation differs from the closed RNA-bound conformation. Indeed, these data confirm that the open conformation observed in the crystal structure exists in solution and is not a lattice artifact. This highlights the advantages of cryo-EM for elucidating protein conformation in solution and provides compelling evidence that RNA and possibly other splicing factors trigger a conformational change in SF3B1.

### PHF5A^R38C^ mutation sensitizes spliceosomes to modulators harboring the C7 cycloheptyl-piperazine

**Figure 4.**
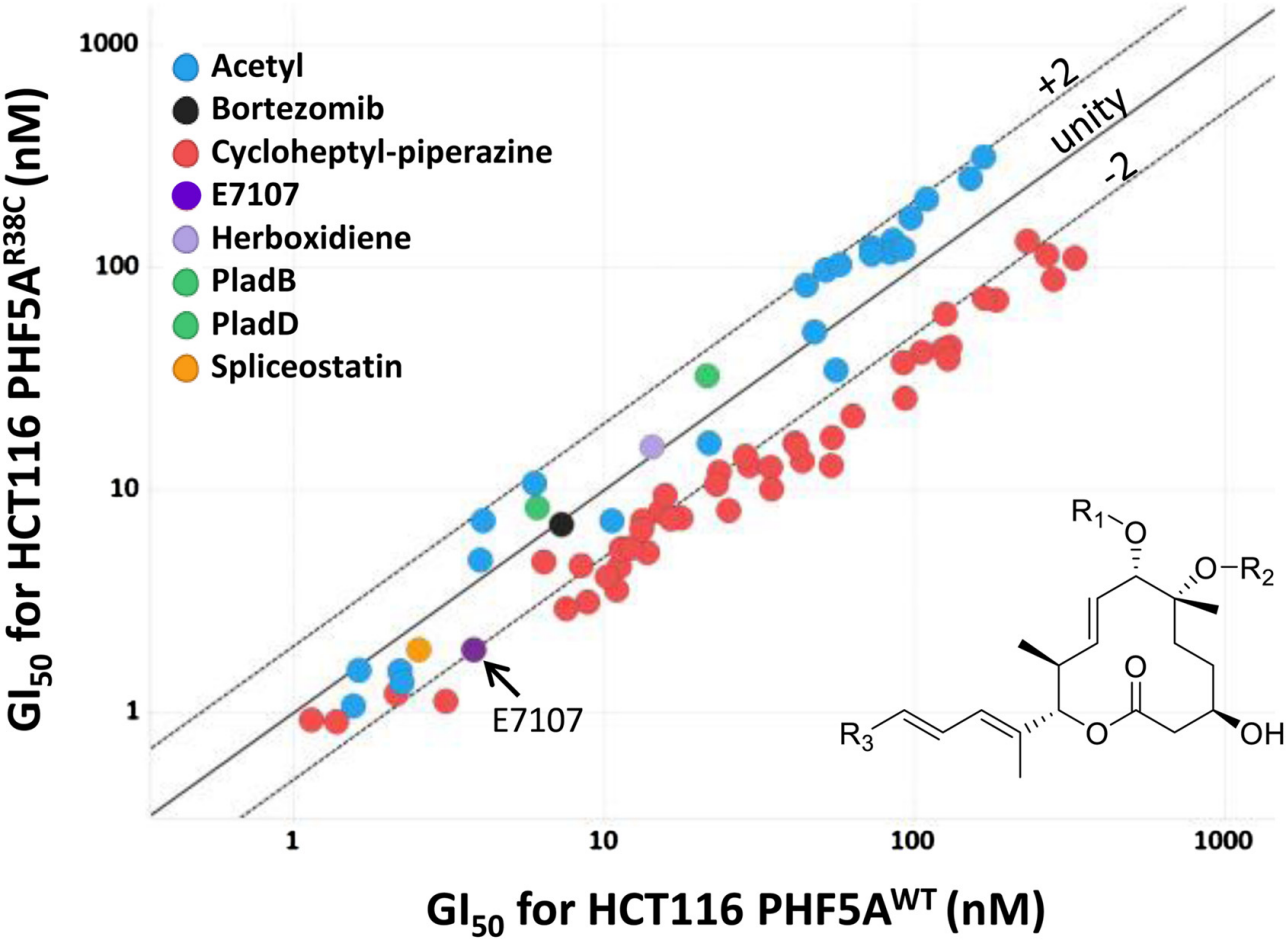
Sensitization phenotype for PHF5A^R38C^. Pladienolides with a cycloheptyl-piperazine at the R1 position (red dots) show a modest sensitization trend with a twofold or greater enhanced potency toward the mutant. Pladienolides with the smaller acetyl group at the R1 position (blue dots) do not show this trend, lying within the range of plus or minus twofold variation. Spliceostatin (orange) and herboxidiene (lavender), which lack the cycloheptyl-piperazine functional group, lie near the line of unity, as does the unrelated control compound bortezomib (black). E7107 is in purple and has twofold sensitization. *n* = 2.

The resistance mutation PHF5A^Y36C^ was shown previously to rescue the growth inhibition phenotype observed in the presence of splicing modulators (Teng et al. 2017). This prompted further exploration and mutagenesis of PHF5A to find other possible resistance mutations (Teng et al. 2017). In the course of our profiling, we serendipitously found that the mutation PHF5A^R38C^ sensitizes cells to certain pladienolide analogs and not to others (Fig. 4; Supplemental Fig. S4). Analysis of the R groups shows a clear trend in which compounds with a bulky cycloheptyl-piperazine substituent at the C7 position exhibit greater potency in the R38C genetic background than the wild type (Fig. 4). Of the 48 compounds in our library with this C7 substituent, 39 compounds showed twofold or greater sensitization. In contrast, we did not observe this trend for compounds that have a smaller acetyl substituent at C7, such as pladienolide B (PladB) and PladD. Of the 23 compounds in our compound collection with the acetyl substituent located at C7, none of them shows a greater than twofold sensitization. This enhanced interaction for the cycloheptyl-piperazine in the R38C background led us to hypothesize that this substituent is in close proximity to PHF5A^R38^. The pose of E7107 in the binding pocket confirms this hypothesis where the cycloheptyl-piperazine approaches PHF5A^R38^ (Fig. 3B). Chemically, this can be rationalized by the disfavored proximity of the two positive charges from the piperazine and the arginine that is ameliorated by the R38C mutation. Of note, other splicing modulators, such as herboxidiene and spliceostatin, did not exhibit this sensitization phenotype (Fig. 4; Supplemental Fig. S4). These compounds have the diene pharmacophore but lack the distinctive piperazine moiety and are therefore consistent with this model.

### Binding pocket interactions

To further validate the pose of the compound, we analyzed the activity of several related chemical probes using the SPA with the purified protein complex (Teng et al. 2017). The SPA is formatted as a competition assay in which the purified protein complex is captured on a bead containing scintillant, incubated with test compounds over a dose response, and then competed off with a fixed concentration of ^3^H-Plad compound. IC_50_ curves are then measured from the resulting light emitted from the bead (Table 1; Supplemental Fig. S4). In addition, we tested the probes in viability assays in two engineered cell lines: HCT116-PHF5A^WT^ and the sensitization line HCT116-PHF5A^R38C^ (Table 1; Supplemental Fig. S5). This facilitates profiling of chemical probe activity in the PHF5A background (wild type and mutant). The relative expression levels were further confirmed by Western blot analysis (Supplemental Fig. S5).

**Table 1.**
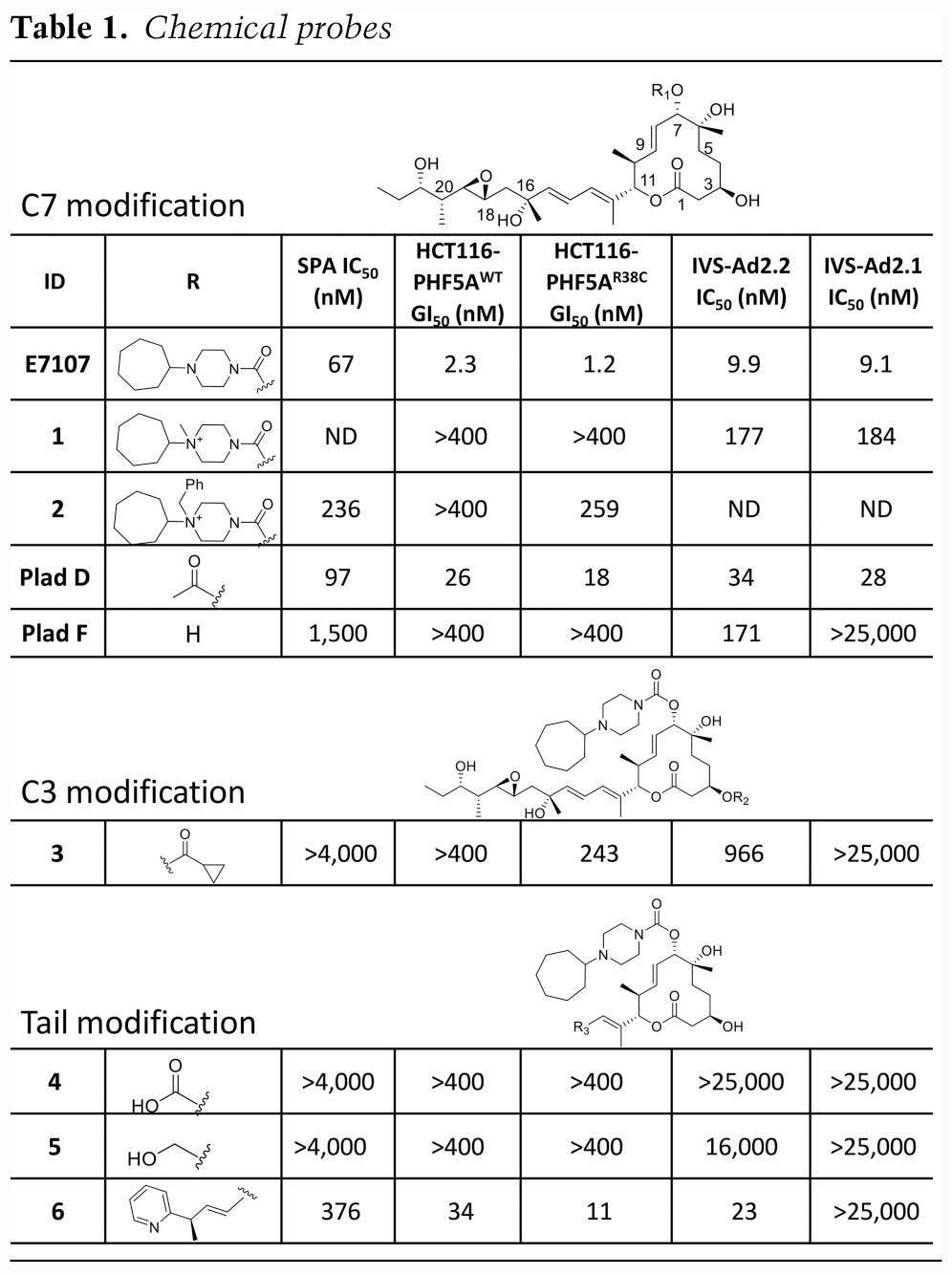
Chemical probes

The cryo-EM structure demonstrates how the cycloheptyl-piperazine is favored at the C7 position, since it picks up hydrophobic interactions with the protein. Incorporating additional charge and molecular weight into this region (compounds 1 and 2) is less favored, and the structure indicates that there is less room to accommodate such larger substituents (Table 1). However, smaller substituents are well tolerated at C7, including the acetyl group seen in PladD. PladD is ∼10-fold less active in the viability assays but is nearly equipotent to E7107 in the SPA binding assay. The OH substituent at C7 (PladF) loses >200-fold potency relative to E7107 in viability and 20-fold in the SPA. We attribute this to the loss of an H-bonding interaction from the acetyl group and PHF5A observed in the structure.

The structure further rationalizes why particular substitutions are not tolerated. For instance, incorporating larger substituents at the C3 position, such as the cyclopropyl carboxylic acid in compound 3, results in ∼1000-fold loss of potency relative to E7107 in viability assays and total loss of activity in the SPA binding assay (Table 1). In the structure, C3 is buried in the pocket, and there is no room to accommodate larger substituents at this position.

Finally, the diene tail at C11 is the critical pharmacophore that occurs in all natural product-based splicing modulators. The structure shows that this key pharmacophore interacts with PHF5A^Y36^. Truncating the diene tail back to an acid or alcohol (compounds 4 and 5) completely eliminates activity despite the presence of the macrocycle and C7 cycloheptyl-piperazine. The structure also reveals that the very end of the diene tail may be more solvent-exposed, which suggests that different tail lengths and compositions may be further tolerated. Indeed, compound 6, which has the pyridine substitution at C16, loses only ∼10-fold potency in viability and sixfold potency in the SPA relative to E7107 (Table 1), suggesting that it is well tolerated within the binding pocket. In summary, the structure–activity relationships (SARs) for these chemical probes are consistent with the binding pose observed in our cryo-EM structure.

### Competition with strong and weak branch point sequence (BPS) substrates

**Figure 5.**
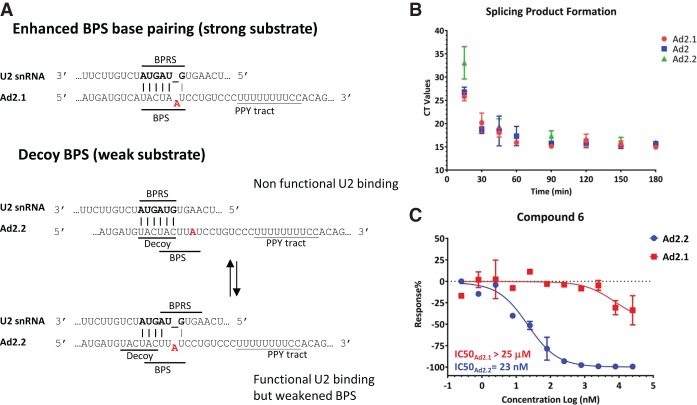
The IVS substrates Ad2.1 (strong) and Ad2.2 (weak). (*A*) Base-pairing logic for IVS substrates. (BPRS) Branch point recognition sequence in U2 snRNA; (BPS) the BPS in the pre-mRNA. The Ad2 gene BPS sequence (UACUUAU) was modified (UACUAAU) to strengthen the U2 base-pairing interactions flanking the BPA, leading to the Ad2.1 substrate (BPA in red). The Ad2.2 sequence has the same BPS as Ad2 but also contains a 5′ decoy BPS sequence (lacking an extrahelical adenine) that can base-pair with the BPRS. The decoy BPS and suboptimal BPS overlap, which gives U2 at least two possibilities for possible base pairing, as indicated. The combination of decoy sequence and suboptimal base-pairing interactions around the BPA weakens this substrate relative to Ad2.1. (*B*) RT-qPCR time-course data showing product formation over time. At the early time points, the threshold cycle value (CT values) required to detect the splicing product is high and then decreases over time as more product is generated. All three substrates show similar splicing efficiencies, and no additional product is formed after 60 min. Approximately 15%–20% substrate conversion occurs under these conditions. (*C*) Representative IC50 curves for compound 6 using the two different substrates (Ad2.1 and Ad2.2). Here, the compound can effectively inhibit splicing of the weak substrate but not the strong.

The cryo-EM structure and activity data for our chemical probes clearly show that the splicing modulators bind in the spliceosome BPA-binding pocket, which immediately suggests that they could directly compete with the RNA substrate. A substrate competitive mechanism of action (MOA) would predict that weaker pre-mRNA substrates would be easier to inhibit than stronger substrates. To date, it has not been possible to evaluate substrate competition using traditional steady-state conditions because splicing reactions are thought to be single-turnover events in vitro, and therefore it is not possible to measure a K_M_ for the pre-mRNA. Nevertheless, by engineering how the pre-mRNA substrate base-pairs with the U2 snRNA in the BPS region, we were able to generate a strong and weak pre-mRNA substrate to build support for a substrate competitive model (Fig. 5A; Supplemental Fig. S6). Specifically, we engineered the strong in vitro splicing substrate Ad2.1 by enhancing the U2 snRNP base-pairing around the BPA found in the Ad2 substrate. We then engineered a weaker substrate, Ad2.2, by adding a decoy BPS lacking an extrahelical adenosine (Ad2.2) (Fig. 5A; Supplemental Vector NTI files; Corrionero et al. 2011). We confirmed that both substrates and the original substrate Ad2 show similar levels of splicing efficiency (Fig. 5B). As a consequence, Ad2.2 has a weaker base-pairing consensus around the BPA and a decoy BPS, and this substrate should be easier to inhibit by splicing modulators than the strong substrate. We then evaluated E7107 and several chemical probes to better understand their mechanism. These data show that under these assay conditions, compounds that are most potent in the SPA-binding assay, such as E7107, PladD, and compound 1, are able to inhibit both the strong and weak substrates equally well. Conversely, compounds that are weaker in the SPA, such as PladF and compound 6, are able to inhibit only the weak substrate Ad2.1 but not the strong substrate Ad2.2 (Fig. 5C; Supplemental Fig. S6). These chemical probes therefore help establish that the degree of complementarity around the BPA and decoy BPSs may contribute in part to the strength of the pre-mRNA substrate. Moreover, the different pre-mRNA substrates in turn help further classify the chemical probes as strong or weak modulators. Importantly, tight binding compounds that are nonselective toward strong and weak substrates in these IVS assays are expected to be more pleiotropic, while weaker binding compounds show preferential inhibition toward short and GC-rich introns (Seiler et al. 2018).[Fig GAD311043FINF6]

**Figure 6. GAD311043FINF6:**
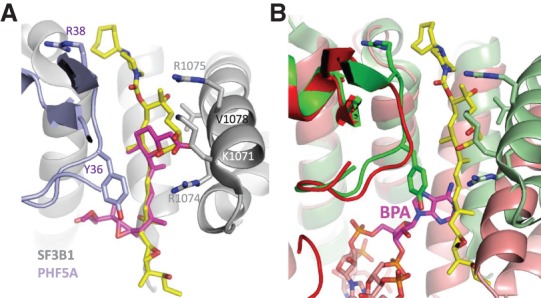
(*A*) Model of how herboxidiene (magenta) may bind based on the cryo-EM structure of E7107. This model was generated by simple overlay of the small molecule crystal structure of herboxidiene and the conserved diene pharmacophore. (*B*) Overlay of SF3B1 from the cryo-EM B^act^ spliceosome structure (red) and E7107 structure (green). The overlay uses PHF5A as a frame of reference and reveals the conformational changes within the BPA pocket upon RNA binding. Moreover, the diene pharmacophore would clash with the BPA, consistent with a model in which the compound locks SF3B1 in an open conformation and competes for the same binding site as the BPA.

## Discussion

### Compound-binding mode relative to resistance and sensitization mutations

Here we report the cryo-EM structure of the SF3b subcomplex at 3.95 Å resolution in complex with E7107. After modeling the protein into the cryo-EM map, there was additional unexplained density located in the BPA-binding pocket, consistent with the presence of E7107. These structural data were then used to orient the compound in the binding pocket. E7107 occupies the channel and places the piperazine in close proximity to PHF5A^R38^. The proximity of these two positive charges would be expected to be slightly disfavored, and our SAR data confirm that E7107 and other Plad analogs with a cycloheptyl-piperazine are more potent in the PHF5A^R38C^ background when the charged residue is neutralized. Furthermore, Plad analogs containing the smaller and uncharged acetyl group are unaffected by this R38C mutation. Thus, these SAR data would be consistent with the piperazine being positioned near PHF5A^R38^. Indeed, the discovery of a mutation that sensitizes the target protein to a drug is highly unusual, and we were able to exploit this serendipitous finding to further validate the orientation of the compound in the cryo-EM map.

The proposed binding mode is also consistent with known resistance mutations. The first reported resistance mutation to E7107 was SF3B1^R1074H^ (Yokoi et al. 2011), and subsequent studies reported additional resistance mutations SF3B1^K1071E^, SF3B1^V1078A/I^, and PHF5A^Y36C^ (Teng et al. 2017). SF3B1^R1074H^ and PHF5A^Y36C^ mutations confer the strongest levels of resistance (>500-fold). Both residues sit deep in the binding pocket on orthogonal sides and are situated adjacent to the diene tail of E7107. It is possible that these two residues are involved in either pi–pi or pi–cation interactions with the diene tail, and mutating them would disrupt these favorable interactions as well as the spatial configuration of the pocket. Mutation of PHF5A^Y36C/S/A/E/R^ confers 100-fold to 5000-fold resistance to E7107 treatment (Teng et al. 2017). The only mutations that confer partial resistance to E7107 are PHF5A^Y36F/W^, which confer fivefold to 10-fold resistance, suggesting that the compound is still able to bind but not as strongly (Teng et al. 2017). These data are all consistent with the binding mode of the compound.

SF3B1^K1071^ and SF3B1^V1078^ are positioned higher in the binding pocket. SF3B1^K1071^ is located at the top of the binding pocket, and mutation to glutamic acid would change the charge complementarity. SF3B1^V1078^ is across from the macrocycle, and mutation to alanine or isoleucine confers a comparatively lower level of resistance (∼20-fold). These changes are more subtle and would reduce the shape complementarity of the binding pocket.

### Compound-binding mode and SAR

Our structure can also be used to rationalize currently available SAR data. First, the common pharmacophore for the natural products pladienolide, herboxidiene, and spliceostatin is the diene tail (Supplemental Fig. S7). The SF3B1^R1074H^ and PHF5A^Y36C^ mutations confer resistance to all three classes, suggesting a common binding mode (Teng et al. 2017). For the pladienolide scaffold, a wide number of substituents is tolerated at the C7 position, consistent with our structure showing that this portion is more surface-exposed. Consistent with our pose, this is also the position where the photo-cross-linking motif was installed and found to cross-link to SF3B3 (Kotake et al. 2007), which is in close proximity. Alignment of the diene pharmacophore for pladienolide and herboxidiene places the carboxylate of herboxidiene near the C3 hydroxyl of the macrocycle (Fig. 6A) and in the immediate vicinity of the electropositive surface defined by SF3B1^K1071^, SF3B1^R1074^, and SF3B1^R1075^. This model agrees with the reported SAR for the herboxidiene analogs that lose about threefold activity when this carboxylate is methylated (Lagisetti et al. 2014). Similarly, we show that modification at this C3 hydroxyl (compound 3) also leads to a significant drop in activity. While some aspects of the SAR can be explained by our structure, other features will require additional structures and higher-resolution data. For instance, the SAR has been reported for the diene tail, showing that removal of the two methyl groups at C16 and C20 results in a large reduction in activity in an in vitro splicing assay (Effenberger et al. 2014), which would suggest that the SAR is very sensitive in this region and may involve direct contacts with the protein. In our structure, however, this tail region is relatively disordered, and such a steep drop-off in activity is difficult to rationalize. Indeed, compound 6 has a shorter diene tail, incorporating a pyridine, and retains potency in all of our assays except the IVS assay with the strong Ad2.2 pre-mRNA substrate. This SAR agrees with our cryo-EM structure that would predict that the SAR near the tail of the molecule would be more tolerant of different substituents. Additional structures and SAR data will provide deeper understanding of this region of the molecule.

### Compound-binding mode and proposed mechanism for splicing inhibition

SF3B1 is critical for BPA recognition. Once the first step of splicing has occurred, the SF3b complex appears to play no further role. In yeast, the ATPase-dependent activity of helicase Prp2 is required for the departure of SF3b and is necessary for the first step of splicing (Lardelli et al. 2010). Evidence is accumulating that departure of the SF3b complex is also required in the human spliceosome during the transition from the B^act^ to the C complex. Proteomics studies show that SF3b proteins are depleted by 70% in the C complex relative to the B^act^ complex (Agafonov et al. 2011). Moreover, the cryo-EM structure of the human C* complex revealed that the SF3b proteins were absent (Zhang et al. 2017), pointing to their departure. These data are all consistent with the paradigm that SF3B1 splicing modulators act early in spliceosome assembly by weakening interactions between the U2 snRNP and the pre-mRNA, likely impeding the formation of the “A complex” (Roybal and Jurica 2010; Corrionero et al. 2011; Folco et al. 2011; Effenberger et al. 2014; Vigevani et al. 2017).

Our cryo-EM structure of the SF3b complex bound to E7107 now provides a structural basis to support their MOA. A closer comparison of the SF3B1 conformation in the reported apo-crystal structure and the RNA-bound structure from the cryo-EM B^act^ structure shows a large conformational change in SF3B1. The apo conformation is in an open conformation, while the RNA-bound form is in a closed conformation (Fig. 1C). Our cryo-EM structure bound to E7107 shows that SF3B1 closely resembles the open conformation. Importantly, this confirms that this open conformation is the relevant solution conformation of the complex and is not influenced by crystal contacts. Moreover, superposition of our E7107-bound structure with the RNA-bound structure from B^act^ shows that the diene tail region of E7107 would clash with the BPA (Fig. 6B). In other words, the binding of the compound and BPA would be mutually exclusive. This suggests a model for a mixed mode of inhibition in which the compound locks the protein in an open and inactive conformation and, moreover, sits in the pocket in such a way as to prevent the binding of the BPA. It is also consistent with a model in which these compounds behave as pre-mRNA substrate competitive inhibitors. In support of this, our chemical probes show differential activity against pre-mRNA substrates with strong and weak BPSs (Fig. 5A). Evidently, the summation of favorable cooperative interactions between the spliceosome and the stronger substrates is sufficient to displace splicing modulators, while weaker substrates are not. Therefore, the degree to which any splicing event is affected depends on the relative strength of the compound, the affinity of the particular pre-mRNA substrate, and their relative concentrations. This explains, at least in part, why these compounds do not behave as universal splicing inhibitors but rather as modulators of certain splice junctions, as exemplified in global RNA-seq studies in which only a subset of junctions is impacted (Seiler et al. 2018). Further structural and biochemical studies will be needed to better characterize the interactions between different pre-mRNA substrates and with different splicing modulators.

Although the potential to perform drug discovery with single-particle cryo-EM is exciting, there are still significant challenges that exist. One of these challenges includes obtaining high-resolution (>3.0 Å) structures and well-defined maps. This limitation hinders elucidating details of the binding pose as well as precise interactions between protein-interacting side chains. Size, symmetry, and intrinsic flexibility are all parameters that play key roles in determining the resolution that can be potentially achieved (Merk et al. 2016). The current resolution limit for the SF3b complex could be affected by the 250-kDa molecular weight and the lack of symmetry displayed by the SF3b complex along with the intrinsic flexibility of SF3B1. Ultimately, the particle orientation preference exhibited by the SF3b complex particles constrained the limit of resolution to 3.95 Å. Our work clearly demonstrates that single-particle cryo-EM can be successfully used to locate the binding site of spliceosome modulators bound to their targets, and the binding pose can be further supported with resistance and sensitization mutations along with compound SAR. This approach can be further extended to the study and optimization of other spliceosome modulators targeting the SF3b complex and may have additional implications in the rational design of therapeutics targeting RNA splicing.

## Materials and methods

### Purification of the recombinant protein complex

The recombinant protein complex was purified essentially as described (Teng et al. 2017). Truncated SF3B1 and full-length SF3B3, PHF5A, and SF3B5 were synthesized and subcloned between the NcoI and EcoRI sites of the pFastBac1 vector. SF3B1 was limited to the HEAT repeat domain from residues 454–1304 and cloned in-frame with an N-terminal Flag tag. Both SF3B3 and SF3B5 contained N-terminal His tags. Viruses were generated and used to coinfect SF21 cells with each virus at a virus:cell ratio of ∼10:1. The cells were harvested 72 h after infection and lysed in 40 mM HEPES (pH 8.0), 500 mM NaCl, 10% glycerol, and 1 mM TCEP. The complex was purified by batch method using nickel-NTA beads followed by Flag beads. The eluent was concentrated and injected on a gel filtration column (superdex 200) in buffer containing 20 mM HEPES (pH 8.0), 300 mM NaCl, 10% glycerol, and 1 mM TCEP. The peak fractions were collected, concentrated to 8 mg/mL, and flash-frozen in liquid N_2_ for storage at −80°C.

### MST

The SF3b subcomplex was first titrated against a fixed concentration (25 nM) of fluorescent label (NTA-647) to determine the optimal concentrations of protein and label for the MST experiment. After 15 min of incubation at room temperature, samples were loaded into Monolith NT.115 capillaries, and MST analysis was performed using a Monolith NT.115Pico. The *K*_D_ for NTA-647 was determined to be 8 nM. For subsequent compound-binding experiments, the SF3b subcomplex bound to NTA-647 was fixed at 10 nM, and the compound was titrated from 50 µM to 3 pM. After 15 min of incubation, samples were loaded into capillaries, and analysis was performed as described previously. A *K*_D_ of 3.6 nM was determined for the interaction with E7107. The same analysis was applied to the PHF5A^Y36C^ mutant complex, and no binding was detected for E7107.

### Cell culture and cell viability assay

Parental HCT116 cells were obtained from American Type Culture Collection and cultured in RPMI 1640 medium supplemented with 10% fetal bovine serum. PHF5A R38C was generated using the Agilent QuikChange II kit and was cloned into pLenti6.3/V5 as described (Teng et al. 2017). The verified clone was used for lentivirus production using X293T cells. Parental HCT116 cells were infected with virus and selected with blasticidin S at 10 µg mL^−1^ for 1 wk. Engineered cell lines were subsequently maintained in the absence of antibiotic. Transgene expression was confirmed by Western blot analysis (Supplemental Fig. S4). For Western blot analysis, α-PHF5A rabbit polyclonal antibody (Protein Tech, 15554-1-AP) was used at 1:1000 dilution in LI-COR buffer, and anti-rabbit secondary antibody (LI-COR) was used at 1:5000 dilution. The Western blot was imaged using an Odyssey V3.0 imager (LI-COR). For CellTiter-Glo analysis of the wild-type and PHF5A^R38C^ cell lines, 500 cells were seeded in each well of a 384-well plate 1 d before compound addition. Dose response was measured in 11-point serial dilutions with a top dose of 10 µM. The percentage of DMSO was controlled throughout, and a DMSO-only control was included. Seventy-two hours after dosing, CellTiter-Glo reagent was added, incubated, and assayed on an EnVision multilabel reader (PerkinElmer). Luminescence values from each treatment were normalized to the average value of the respective DMSO control. The dose response curve plots were generated in GraphPad Prism, and GI_50_ values were calculated from nonlinear regression analysis. GI_50_ values for the wild-type and R38C mutant cell lines were plotted in Spotfire.

### In vitro splicing reaction

The Ad2-derived (Pellizzoni et al. 1998) sequence was cloned into the pGEM-3Z vector (Promega) using 5′ EcoRI and 3′ XbaI restriction sites. The Ad2 variant (Ad2.2) (Seiler et al. 2018) was cloned into pcDNA3.1(+) vector (Promega) using the same restriction sites. The plasmids were linearized using XbaI and used as DNA templates in the in vitro transcription reactions. The FtzΔi intron-less plasmid (Luo and Reed 1999) was linearized using EcoRI. All RNAs were in vitro transcribed and then purified using MegaScript T7 and MegaClear kits, respectively (Invitrogen). For splicing reactions used with the Ad2.2 substrate, 1-µL reactions were prepared using 8 µg of nuclear extracts prepared from HeLa S3, a saturating excess of 2 ng of pre-mRNA substrate, 0.2 ng of FTZΔi, and varying concentrations of compounds or DMSO. Under these conditions, we typically observed ∼15%–20% conversion of substrate to product. After 15 min of preincubation at 30°C, 1 µL of splicing activation buffer (0.5 mM ATP, 20 mM creatine phosphate, 1.6 mM MgCl_2_) was added, and the reactions were incubated for 90 min at 30°C. The reactions were then quenched with 13 µL of DMSO, and 25 nL was used for RT-qPCR. RT-qPCR reactions were prepared using the TaqMan RNA-to-C_T_ 1 step kit (Life Technologies), RNA from splicing reactions, and Ad2 junction (forward, ACTCTCTTCCGCATCGCTGT; reverse, CCGACGGGTTTCCGATCCAA; and probe, CTGTTGGGCTCGCGGTTG) and Ftz (forward, TGGCATCAGATTGCAAAGAC; reverse, ACGCCGGGTGATGTATCTAT; and probe, CGAAACGCACCCGTCAGACG) mRNA primer–probe sets. GraphPad Prism was used for nonlinear regression curve fitting of the spliced product formed and normalized to the untreated/DMSO sample.

### SPA

Batch immobilization of the anti-Flag antibody (Sigma) to the anti-mouse PVT SPA scintillation beads (PerkinElmer) was prepared as follows. For every 1.5 mg of beads, 10 µg of antibody was prepared in 150 µL of PBS. The antibody–bead mixture was incubated for 30 min at room temperature and centrifuged at 18,000*g* for 5 min. One-hundred-fifty microliters of PBS was used to resuspend every 1.5 mg of antibody–bead mixture. The aforementioned mini-SF3b complex was tested for ^3^H-labeled pladienolide probe binding (Kotake et al. 2007). One-hundred microliters of binding reactions was prepared with 50 mL of bead slurry and 0 or 50 nM protein in buffer (20 mM HEPES at pH 8, 200 mM KCl, 5% glycerol). The mixture was incubated for 30 min, and varying concentrations of ^3^H-labeled pladienolide probe were added. The mixture was incubated for 30 min, and luminescence signals were read using a MicroBeta2 plate counter (PerkinElmer). Compound competition studies were performed with the wild-type mini-SF3b complex. One-hundred microliters of binding reactions was prepared with 50 µL of bead slurry, 25 nM protein in buffer, and compounds at varying concentrations. After 30 min of preincubation, 10 nM ^3^H-labeled pladienolide probe was added. The reactions were incubated for 30 min, and luminescence signals were read.

### Generation of the drug-bound SF3b complex

To generate the drug-bound SF3b complex, fivefold molar excess of the small molecule E7107 was incubated with the protein for 30 min on ice. The complex was then buffer-exchanged to prepare the EM specimen. We did not completely remove the glycerol from the buffer, as the protein would aggregate severely under cryogenic conditions. Instead, we gradually decreased the concentration of glycerol to 2%, which is tolerable for cryo-EM specimen preparation, while the protein still maintained good behavior.

### EM specimen preparation and data acquisition

Aliquots of 4.0 μL of purified SF3B complex at a concentration of ∼1 mg/mL were placed on glow-discharged holey carbon grids (300 mesh, Quantifoil, Au R1.2/1.3). Grids were blotted for 3.0 sec and flash-frozen in liquid ethane cooled by liquid nitrogen using the Vitrobot Mark IV (FEI). Grids were subsequently transferred to a Titan Krios (FEI) electron microscope that was equipped with a Gatan GIF quantum energy filter operating at 300 kV with a nominal magnification of 210,000×. The pixel size was 0.661 Å per pixel. Zero-loss movie stacks were collected automatically using eTas (developed by Xueming Li at Tsinghua University) or AutoEMation (Lei and Frank 2005) with a slit width of 20 eV on the energy filter and a defocus range from −1.2 to −2.2 µm. Each stack was exposed in counting mode for 8 sec with an exposure time of 0.15 sec per frame, resulting in a total of 53 frames per stack. The total dose rate was ∼55 e^−^/Å^2^ for each stack. The stacks were then motion-corrected with MotionCor2 (Zheng et al. 2017) as well as dose weighting (Grant and Grigorieff 2015b). The defocus values were estimated with Gctf (Zhang 2016).

### Image processing

A diagram of the procedures for data processing is in Supplemental Figure S2. A total of 3371 good micrographs was selected manually, from which a total of 900,715 particles was automatically picked using RELION 2.0 (Scheres 2012a,b, 2015; Kimanius et al. 2016). After 2D classification, a total of 503,680 good particles was selected and subjected to three-dimensional (3D) autorefinement with an initial model obtained from the atomic model (PDB ID 5IFE) with e2pdb2mrc.py from the EMAN2 software package (Tang et al. 2007). The resolution after processing was 5.4 Å. The Euler angle distribution of the 3D autorefinement showed orientation bias. In order to overcome this orientation preference, the data set was filtered by a Python script (eulerbalancing.py) that shaved the particle number in each direction according to the average particle number. The particle number shaving was performed several times, with a local soft mask excluding the flexible β-propeller domains of SF3B3. The data set was first shaved to five times that of the average particle number, resulting in 415,617 particles. After 3D autorefinement, the data set was further shaved to 50% or five times the average particle number, resulting in a small or large data set containing 154,543 or 385,180 particles, respectively. The large data set was further 3D-autorefined, with the 3D reconstruction map obtained from the small data set 3D autorefinement as the initial model. The resolutions after processing of small and large data sets were 4.7 Å and 4.1 Å, respectively. After 3D autorefinement, the large data set was further subjected to several cycles of local angular searching 3D classification with three or four classes, with the output from different global angular searching iterations of the 3D autorefinement as input. A total of 280,768 good particles was selected from the local angular search 3D classification and combined. Next, these particles were subjected to a local angular search 3D autorefinement with a soft mask applied, resulting in a 3D reconstruction map with a resolution of 4.0 Å after processing. The data set was further subjected to random-phase 3D classification (Grant and Grigorieff 2015a) to remove bad particles. The final resolution of the 3D autorefinement was 3.95 Å after processing, during which the option ampl_corr was applied. The final particles number was 241,288. The binning factor of the data set for final 3D refinement was 2. The resolution was estimated with the gold-standard FSC 0.143 criterion (Rosenthal and Henderson 2003) with the high-resolution noise substitution method (Chen et al. 2013). All of the 2D and 3D classifications and autorefinements were performed with RELION 2.0.

### Atomic model refinement

The starting model for refinement was the apo-crystal structure 5IFE. Real space refinement using secondary structural restraints and geometry restraints was performed in PHENIX. Manual adjustments were made in COOT following a final round of real space refinement in PHENIX. The small molecule crystal structure coordinates of E7107 were provided courtesy of Eisai, Inc. The geometry restraints for the ligand were generated using Schrodinger and modeled in COOT.

### Data

The high-resolution map and modeled coordinates have been deposited in the Electron Microscopy Data Bank (EMDB) and PDB with the accession numbers 6915 (EMDB) and 5ZD5 (PDB).

## Supplementary Material

Supplemental Material
