# Supplementary material for: The cryo-EM structure of the SF3b spliceosome complex bound to a splicing modulator reveals a pre-mRNA substrate competitive mechanism of action
Source: Genes Dev. 2018 Feb 1;32(3-4):309–20. doi: 10.1101/gad.311043.117 (PMC5859971; doi:10.1101/gad.311043.117)
Supplement: Supplemental Material [file supp_32_3-4_309__index.html]

Supplemental Material 

# The cryo-EM structure of the SF3b spliceosome complex bound to a splicing modulator reveals a pre-mRNA substrate competitive mechanism of action

## Supplemental Material

- Supplemental\_Data.pdf
