# Supplementary material for: The cryo-EM structure of the SF3b spliceosome complex bound to a splicing modulator reveals a pre-mRNA substrate competitive mechanism of action
Source: Genes Dev. 2018 Feb 1;32(3-4):309–20. doi: 10.1101/gad.311043.117 (PMC5859971; doi:10.1101/gad.311043.117)
Supplement: Supplemental Material [file supp_32.3-4.309_Supplemental_Data.pdf]

Supplemental Figure 1

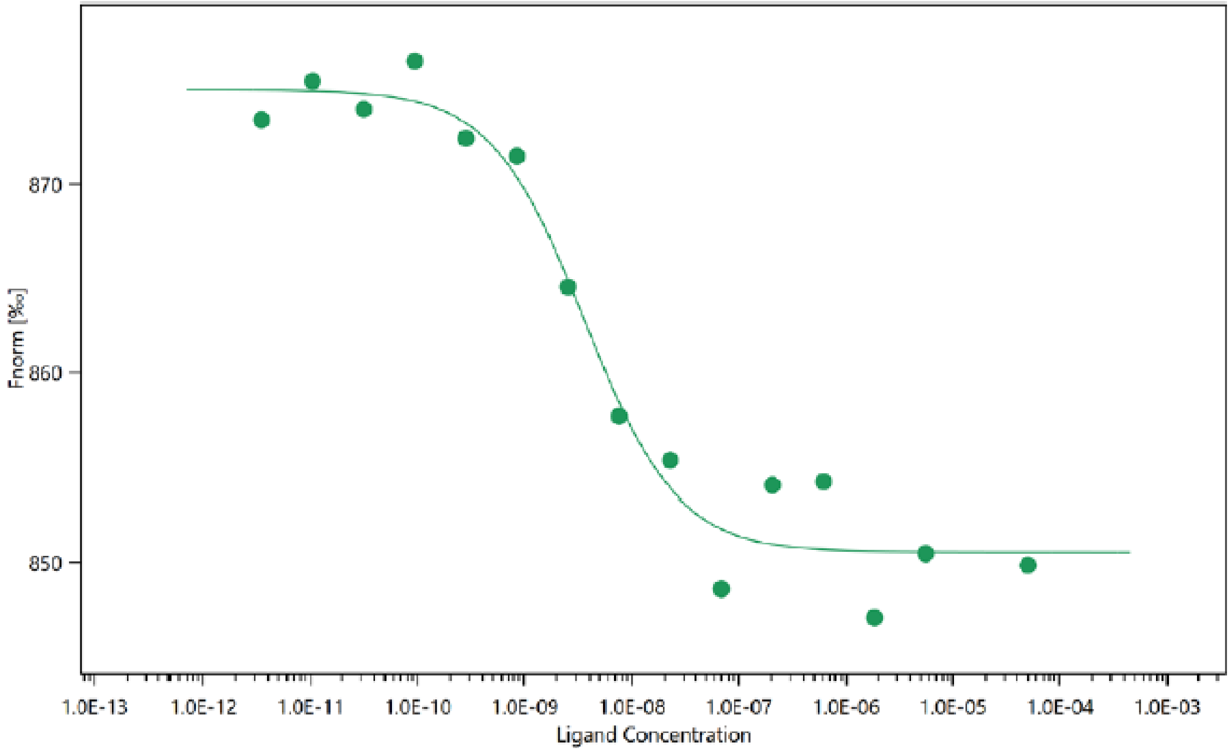

Binding of E7107 to SF3b 4-protein complex by MicroScale Thermophoresis (MST). The complex is labeled with the fluorescent dye NTA-647 and kept constant at 10 nM. E7107 was titrated and a K_D of 3.6 nM was determined for the interaction.

Supplemental Figure 2

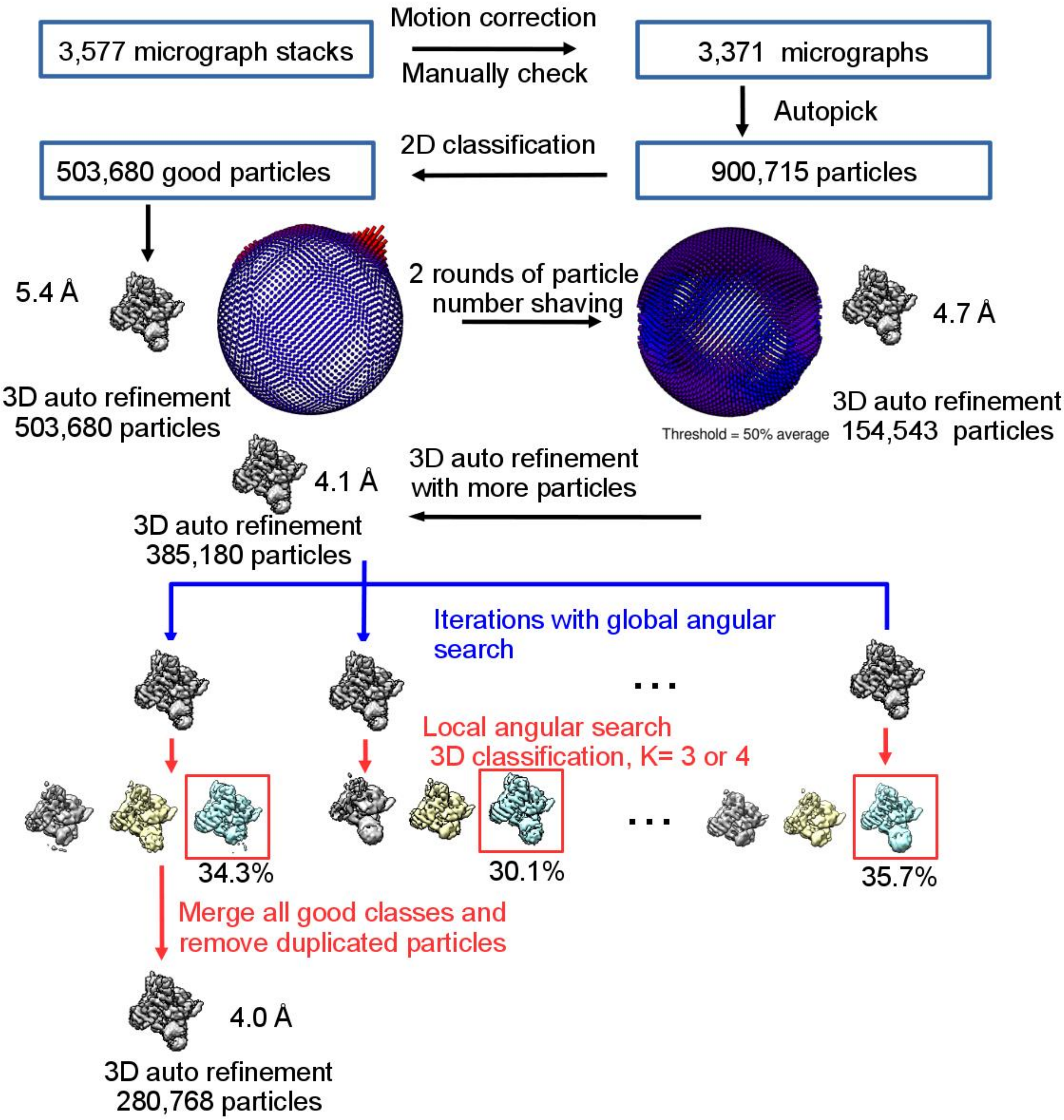

Figure S2. A flowchart representing the cryo-EM data processing of SF3b. Details can be found in the Materials and Methods.

Table S1. Table S1. Cryo-EM data collection and refinement statistics

**Data collection**	
EM equipment	FEI Titan Krios
Voltage (kV)	300
Detector	Gatan K2 Summit
Pixel size	0.661
Electron dose ($e^-/Å^2$)	55
Defocus range (μm)	-1.2 - 2.2
Number of collected micrographs	3,577
Number of selected micrographs	3,371
**Reconstruction**	
Software	RELION 2.0
Number of used Particles	241,288
Symmetry	P1
Resolution (Å)	3.95
Map sharpening B-factor ($Å^2$)	-150
**Refinement**	
Software	PHENIX
Cell dimensions	
a=b=c (Å)	264.4
$\alpha=\beta=\gamma$ (°)	90
R.m.s deviations	
Bonds length (Å)	0.01
Bonds Angle (°)	1.28
Ramachandran plot statistics (%)	
Preferred	89.45
Allowed	10.46
Outlier	0.09
**MolProbity**	
Rotamer Outliers	0.97%
C-beta deviations	0.0
Clashscore	11.28
MolProbity score	2.13

Supplemental Figure 3

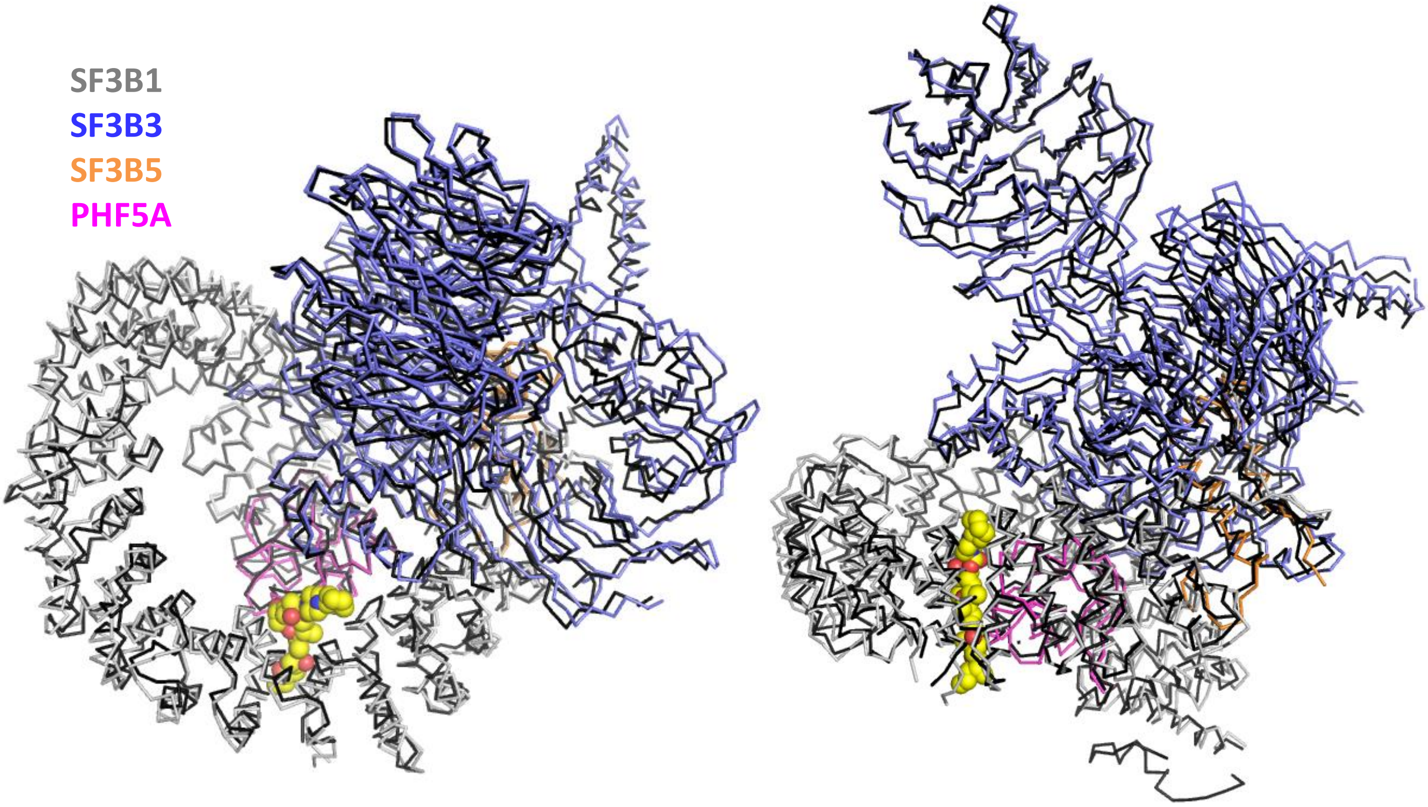

An overlay of the apo crystal structure and the cryo-EM structure. The apo crystal structure is rendered in black (PDB 5IFE) with our ligand bound cryo-EM structure HEAT repeats of SF3B1 shown in gray, PHF5a in magenta, SF3B3 in blue, and SF3B5 in orange. The compound is illustrated in space filling mode. E7107 is rendered as space filling spheres and colored by atom type. The apo and compound-bound structures are very similar with RMSD for Cα atoms < 1.5 Å.

Supplemental Figure 4

SPA competition assay

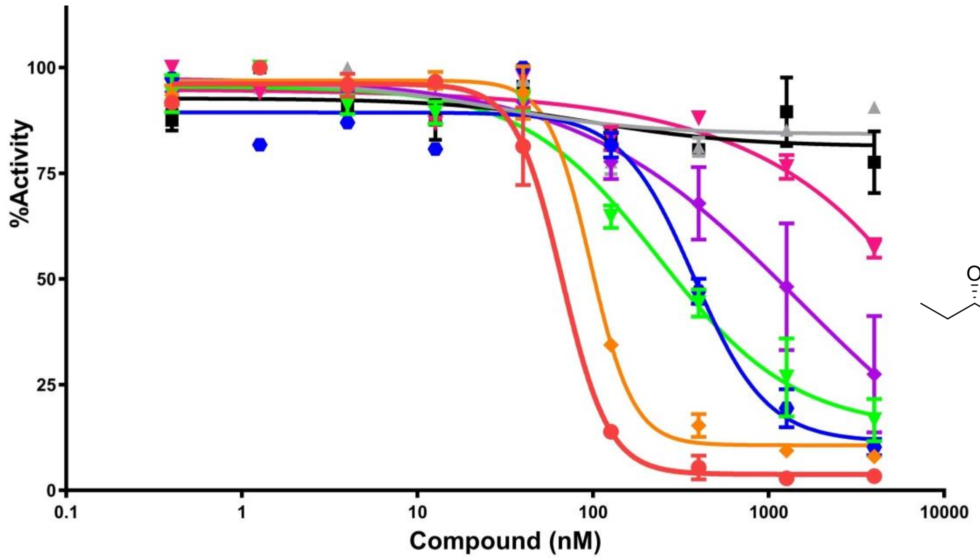

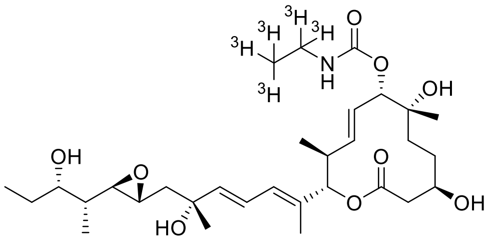

The Scintillation proximity assay (SPA) competition assay with 10 nM ^{3}H tracer for different Pladienolide analogs binding to the 4-protein SF3b complex. Error bars represent STD for 2 or more measurements. Red = E7107, Orange = PladD, Purple = PladF, Green = compound 2, red = compound 3, gray = compound 4, black = compound 5, blue = compound 6.

Supplemental Figure 5

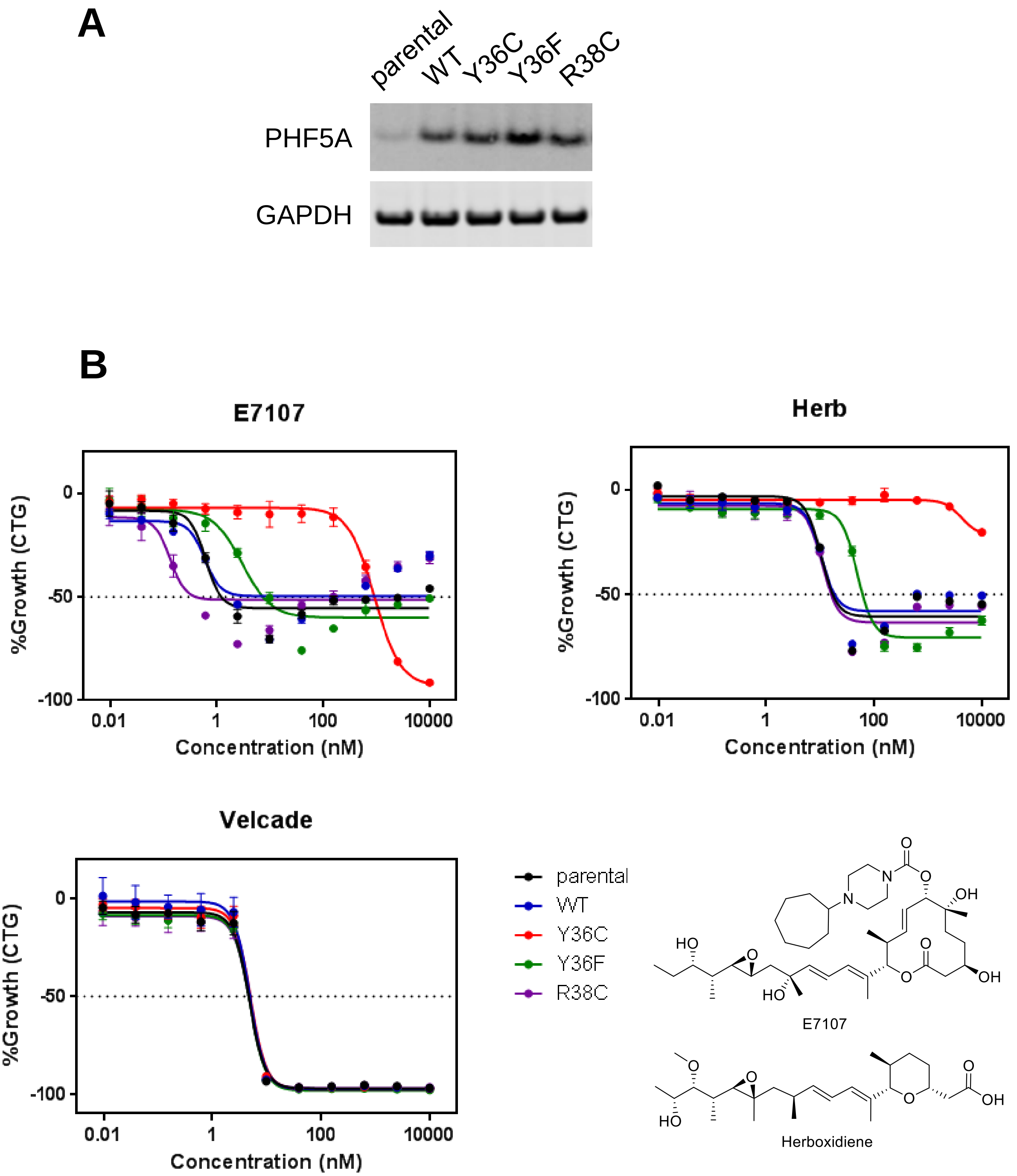

Figure S5. Viability assay data. (A) Western blot showing similar levels of PHF5A over expression in engineered HCCT116 cell lines that were used for viability studies. (B) Representative viability curves in HCT116 cell lines overexpressing PHF5A WT and mutants. Y36C and Y36F confer different degrees of resistance for both E7107 and herboxidiene. The R38C mutation leads to a sensitization for E7107 but not herboxidiene. Velcade (bortezomib) kills all of the cell lines at the same concentration irrespective of the PHF5A mutation status. Chemical structures of E7107 and Herboxidiene are illustrated.

Supplemental Figure 6

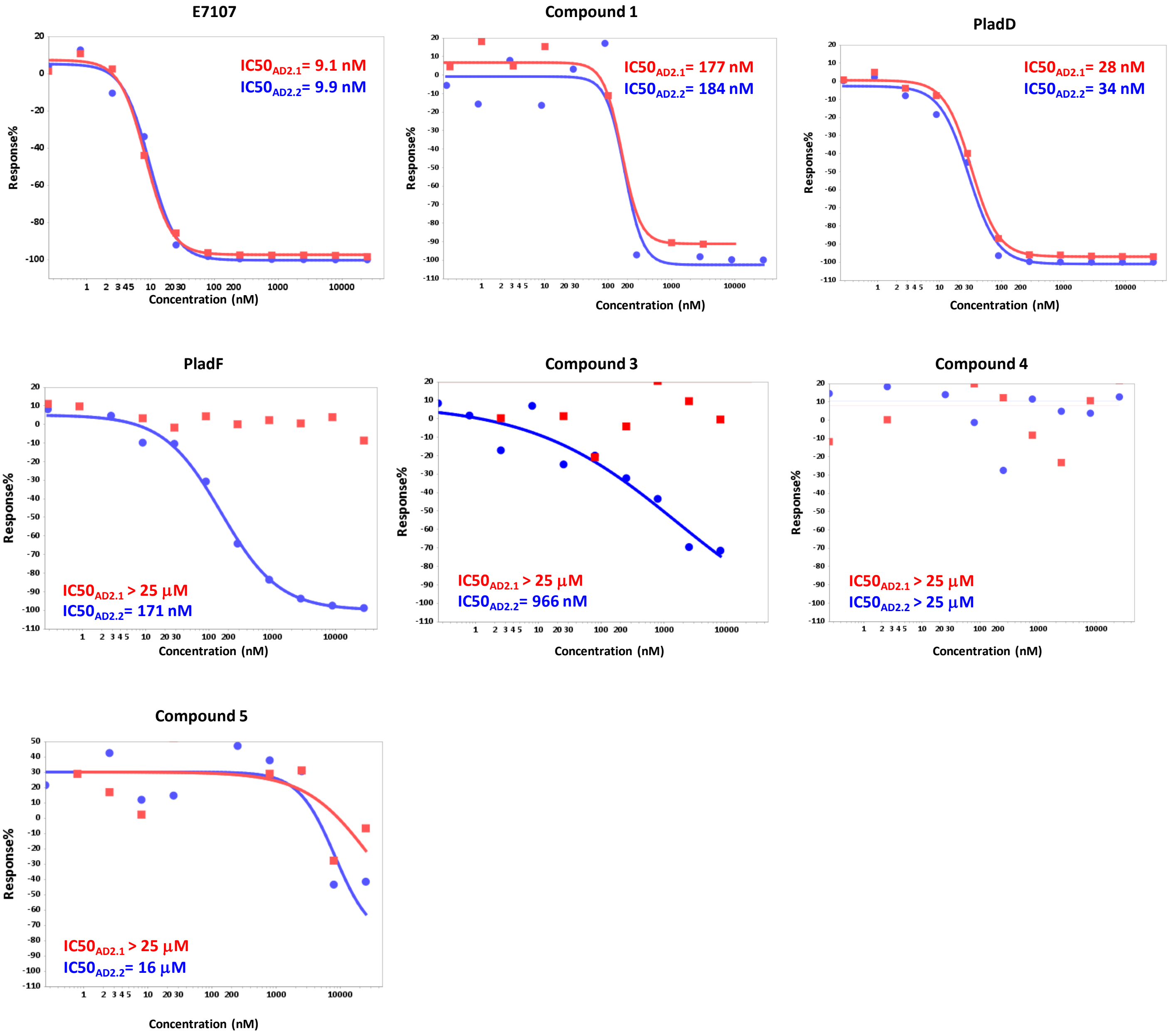

Figure S6. The chemical probes in this study were evaluated in the IVS assay using the two different substrates Ad2.1 and Ad2.2. Differential behavior is seen depending on the strength of the modulator. Potent modulators inhibit both the strong and weak substrates. Less potent modulators (PladF above and compound 6, shown in Figure 5C) inhibit weak substrates but are inactive against strong substrates.

Supplemental Figure 7

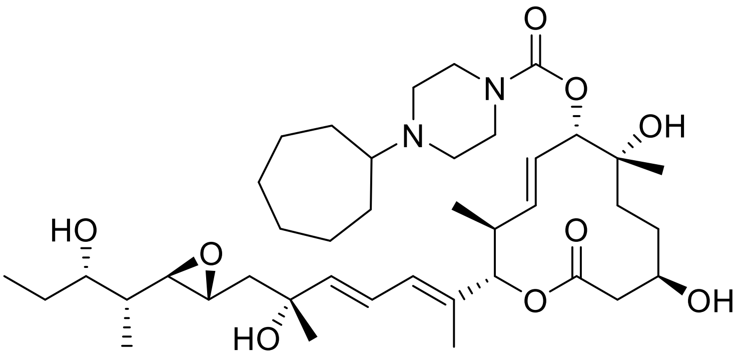

E7107

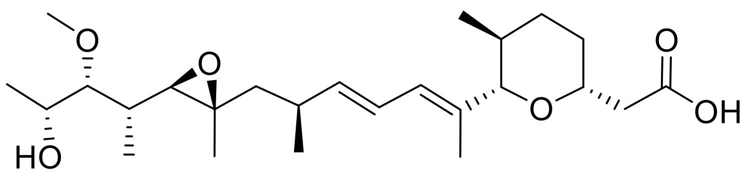

Herboxidiene

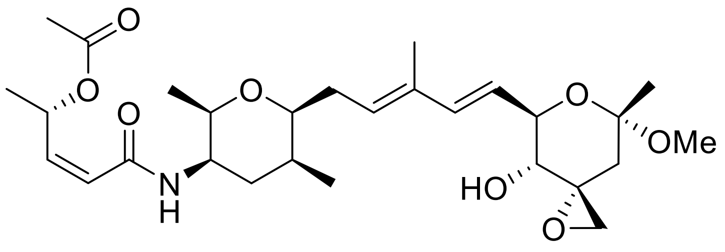

Spliceostatin A

Figure S7. Chemical structures of splicing modulators E7107, herboxidiene and spliceostatin A.
